# Influence of Extraction Techniques and Solvents on the Antioxidant and Biological Potential of Different Parts of *Scorzonera undulata*

**DOI:** 10.3390/life13040904

**Published:** 2023-03-29

**Authors:** Sourour Idoudi, Khadija Ben Othman, Jalloul Bouajila, Audrey Tourrette, Mehrez Romdhane, Walid Elfalleh

**Affiliations:** 1 Energy, Water, Environment and Process Laboratory, (LR18ES35), National Engineering School of Gabes, University of Gabes, Gabes 6072, Tunisia; sourour.idoudi@univ-tlse3.fr (S.I.); khadija_benothman@yahoo.fr (K.B.O.); mehrez.romdhane@univgb.tn (M.R.); 2 Research Unit Advanced Materials, Applied Mechanics, Innovative Processes and Environment, UR22ES04, Higher Institute of Applied Sciences and Technology of Gabes (ISSATG), University of Gabes, Gabes 6072, Tunisia; 3 CIRIMAT, Faculté des Sciences Pharmaceutiques, Université de Toulouse, 35 Chemin des Maraîchers, 31400 Toulouse, France; audrey.tourrette@univ-tlse3.fr; 4 Laboratoire de Génie Chimique, Université de Toulouse, CNRS, INP, UPS, F-31062 Toulouse, France; jalloul.bouajila@univ-tlse3.fr

**Keywords:** *Scorzonera undulata*, extraction methods, solvents, chemical composition, LC-ESI–MS, GC-MS, biological activities

## Abstract

The genus *Scorzonera* has various medicinal values. Species belonging to this genus were traditionally used as drugs or in food. The current study aimed to determine the phytochemical composition, antioxidant activity, and biological properties of the tuber, leaf, and flower of *Scorzonera undulata* extracts, collected from the southwest of Tunisia. Phenolic compounds from the three parts were extracted using two solvents (water and ethanol) and two extraction techniques (maceration and ultrasound). The total phenolic content was measured by the Folin–Ciocalteu assay. Furthermore, the chemical composition of *Scorzonera undulata* extract was also investigated by the LC-ESI–MS method using phenolic acid and flavonoid standards. The variation of the extraction methods induced a variation in the real potentialities of the three parts in terms of bioactive molecules. However, the aerial part of *S. undulata* (leaves and flowers) showed, in general, the highest phenolic contents. Twenty-five volatile compounds have been detected by GC-MS in *S. undulata* extracts; among them, fourteen were identified before derivatization. The DPPH test showed that the aerial part of the plant has a higher antioxidant activity compared to the tuber (25.06% at 50 µg/mL for the leaf ethanolic extract obtained by ultrasound extraction). For most biological activities (anti-Xanthine, anti-inflammatory, and antidiabetic (alpha-amylase and alpha-glucosidase)), the aerial parts (flowers and leaves) of the plant showed the highest inhibition than tubers.

## 1. Introduction

The genus *Scorzonera* of the *Asteraceae* family includes about 200 species. It is widespread in temperate and subtropical regions of Eurasia and North Africa [1]. This genus is represented by perennial herbs with a caudex or tuber, and it is rarely represented by biennials or dwarf subshrubs. The leaves are significantly longer than they are broad and are usually whole [2]. It is characterized by the production of multiflorous, homogamous, and ligulate flower heads. *Scorzonera* has great economic importance; several species belonging to this genus were used in food, including *Scorzonera hispanica* [3] and *Scorzonera austriaca* [4]. They are also employed for their therapeutic potential against colds, diarrhea, and fever caused by bacterial and viral infections, pulmonary disease, parasitic diseases, wounds, and gastrointestinal disorders [5], due to their richness in bioactive compounds such as polyphenols, flavonoids, dihydroisocoumarins, triterpenes, sesquiterpenes, lignans, stilbenes, and others [6].

Among these species, *Scorzonera undulata* is a plant that grows in hills, sandy clay alluvium, and pastures. The primary usage of *S. undulata* is as food. In Tunisia, the tubers are consumed either raw or boiled in water and are appreciated for their sweetness. They are also employed in the preparation of a decoction for its purgative properties. Burns might be effectively treated using the ashes of burned *S. undulata* tubers [7].

Previous studies evaluated the effects of solvents and extraction techniques on the polyphenols and biological activities of plant extracts. The choice of solvent and methods for polyphenol extraction is affected by the plant material, the polarity of the molecules to be extracted, and the intended level of extract purity [8]. There are many techniques for extracting phenolic compounds from plants. Maceration is a conventional extraction method based on the immersion of plant parts in a solvent (water, ethanol, etc.) for a few hours to several days at room temperature. During the extraction process, effectiveness is increased using repetitive maceration, grinding of plant material, high temperatures, and agitation. The main advantages of maceration are its simplicity and low cost, furthermore it does not require complex equipment [9,10]. Over the past three decades, UAE (ultrasound-assisted extraction) has been widely used to achieve significant extraction efficiency in the food and pharmaceutical industries [11]. The process is based on the cavitation occurrence. A succession of compressional and rarefaction waves is applied in the transmission of ultrasound in liquid systems. These waves have the potential to cause the formation of cavitation bubbles in fluids. Pressure and temperature damage the plant cell walls, making bioactive chemicals more easily extracted. Temperature, frequency, duration, type, and volume of solvents, moisture content, and particle size of plant material all have an important impact on the extraction of bioactive compounds [12,13].

A Sari et al. [14] demonstrated that an ethyl acetate extract of *Scorzonera hieraciifolia* roots revealed the highest concentration of phenolic content and antioxidant effect compared to the solvents (chloroform, ethyl acetate, and n-butanol). The effect of extraction solvents and methods was also studied by Llorent-Martínez et al. [15], and the result showed that the chemical composition of plant extracts was dependent on the extraction method and solvent polarity. The study of Athmouni et al. [6] focused on the effect of solvents with different polarities on the phenolic content and antioxidant activity of *S. undulata* roots. The results showed that the solvent combinations significantly impacted the extraction of phenolic compounds and the antioxidant activity of *S. undulata*. On the other hand, the chemical composition and the antimicrobial activities of the essential oil extracted from *S. undulata* aerial parts and capitula were determined; in total, 36 compounds were detected by GC-MS, of which the main molecules were hexadecanoate, methyl linolenate, and heneicosane. The essential oil of *S. undulata* subspecies *deliciosa* showed important antibacterial activity against both gram-positive and gram-negative bacteria, but no antifungal activity was found [7].

The present study aimed to describe the chemical composition of *S. undulata* parts (tubers, leaves, and flowers) extracts by LC-ESI–MS and GC-MS, the antioxidant activity (DPPH), as well as their biological activities: antidiabetic (alpha-amylase and alpha-glucosidase), anti-inflammatory (5-LOX), and anti-xanthine oxidase (anti-XOD).

## 2. Materials and Methods

### 2.1. Plant Material

Fresh samples of *S. undulata* parts (flowers, leaves, and tubers) were collected at an altitude of 66 m from Gabes, Tunisia, on 20 February 2021 (33°48′22″ N 10°4′9″ E). The plant materials were identified by Dr. Hédia Hannachi. After air-drying, the plants were washed, dried, and stored in darkness at room temperature.

### 2.2. Polyphenolic Extract Preparation

The secondary metabolites were extracted from the harvested parts using two different techniques: cold maceration, a conventional extraction method, and ultrasound, an environmentally friendly method.

#### 2.2.1. Maceration

Dried powdered samples of *S. undulata* parts (1 g) were extracted with 20 mL of two solvents with different polarities (ethanol and water) for 24 h at ambient temperature and pressure with medium agitation. The homogenate was filtered using Wattman paper, and then the solvents were evaporated by a rotary evaporator (Staufen, Germany; IKA; RV 10 auto V) under a vacuum at 35 °C.

#### 2.2.2. Ultrasound

The ultrasound extraction was applied using the ultrasonic bath (clean-120hd) for 30 min at a temperature of 30 °C. the mixture was filtered using Wattman paper, then the solvents were evaporated by a rotary evaporator (Staufen, Germany; IKA; RV 10 auto V) under a vacuum at 35 °C.

### 2.3. Total Phenolic Content (TPC)

The polyphenolic content was evaluated by Folin-Ciocalteu methods as described by Ben Khadher et al. [16] with some modifications. In total, 100 µL of Folin-Ciocalteu reagent (0.2 N) and 20 µL of each extract (1 mg/mL) were mixed in a 96-well microplate. After 5 min at room temperature, 80 µL of Na_2_CO_3_ at a concentration of 75 g/L was added, and the mixture was then re-incubated for 15 min in darkness and at room temperature. The absorbance was measured with a microplate reader at a wavelength of 765 nm. The total phenolic content (TPC) was expressed as mg of gallic acid equivalents per g of dry weight (mg GAE/g DW).

### 2.4. Gas Chromatography-Mass Spectrometry (GC-MS) Analysis

The volatile compounds of *S. undulata* extracts were identified using the procedures of Rahmani et al. [17]. For analysis, the ethanol samples were prepared in their principal solvents, wherever the water samples were prepared in methanol, at a concentration of 5 mg/mL. The analysis was done on an Agilent 6890 gas chromatograph coupled to a 5975-mass detector. A volume of 1 μL of each extract was injected by the 7683 B autosampler with the use of a DB-5 MS fused silica capillary column (Supelco, Sigma-Aldrich, St. Louis, MI, USA) (30 m × 0.25 mm inner diameter, film thickness 0.25 μm). Helium (99.99% purity) was operated as a carrier gas at a flow rate of about 0.8 mL/min. Mass spectra have been registered at 70 eV with the use of an ion source temperature held at 310 °C and a transfer line heated at 320 °C. Every record of the acquisition was made in full scan mode (50–400 amu). Compounds were identified by comparison of their mass spectra with those obtained in NIST 08.

#### Derivatization Method

Using a modified method described by Rahmani et al. [17]. The various extracts were prepared in acetonitrile at a concentration of 5 mg/mL, and 60 μL of BSTFA (*N*, *O*-bis(trimethylsilyl)trifluoroacetamide) was added to the extracts (340 μL, 5 mg/mL) and mixed in a 2 mL vial. Then, the mixture was aerated with bubbling nitrogen and shaken for 30 s. The reaction mixture was maintained at 40 °C for 30 min. Finally, 2 µL of each derivatized solution was injected into the GC-MS and analyzed.

The data was collected and analyzed using the software Xcalibur. NIST 8 was used to identify the extracted compounds by comparison of the mass spectra.

### 2.5. Liquid Chromatography-Electrospray Ionization-Tandem Mass Spectrometry (LC-ESI–MS) Analysis

The LC-MS analysis of *S. undulata* extracts was performed in an LC–MS-2020 quadrupole mass spectrometer installed with an electrospray ionization source (ESI) and programmed to negative ionization mode. Separation was accomplished using an Aquasil C18 guard column (10 mm × 3 mm, 3 μm) and an Aquasil C18 (150 mm × 3 mm, 3 μm) (Thermo Electron, Dreieich, Germany) (Thermo Electron). A high-speed liquid chromatography system, including a binary pump system (LC-20AD XR), a column oven (CTO-20AC), a degassing system (DGU-20A 3R), and an autosampler (SIL-20AC XR), were connected online to the mass spectrometer (Shimadzu, Kyoto, Japan). The mobile phase was made up of 0.1% formic acid (*v*/*v*) in H_2_O as solvent A and 0.1% formic acid (*v*/*v*) in methanol as solvent B. Between each run, there was a regulated equilibration interval of 5 min. The column temperature was set at 40 °C, the injection volume was 5 µL, and the mobile phase flow rate was 0.4 mL/min. Spectra were analyzed using Shimadzu LabSolutions LC-MS software while being watched in SIM (selected ion monitoring) mode. The mass spectrometer was used in negative ion mode with a block source temperature of 400 °C, a dissolving line temperature of 250 °C, a 1.5 L/min nebulizing gas flow, a 12 L/min dry gas flow rate, a capillary voltage of −3.5 V, and the whole scan spectra from 50 to 2000 Da. The phenolic compounds of *S. undulata* extracts were identified by corresponding the retention times and mass spectra of the molecule with those of the real standards.

### 2.6. Antioxidant Activity (DPPH Radical Scavenging Activity)

The DPPH scavenging activity of *S. undulata* extracts was measured according to Dawra et al. [18]. In brief, 20 μL of various diluted plant extracts (50 µg/mL) were added to 180 μL of DPPH methanolic solution (0.2 mM), using a 96-well microplate. Vitamin C, at a concentration of 4 µg/mL, was used as a reference. After an incubation of 25 min at 25 °C, the absorbance was measured at 524 nm using a microplate reader. The following formula was used to determine the percentage of ability to scavenge the DPPH radical:

(1) $$\mathrm{Scavenging}\;\mathrm{activity}\;\left(\%\right)=\frac{A_{\mathrm{control}}-{\;A}_{\mathrm{sample}}}{A_{\mathrm{control}}}\times 100$$

where A_control_ is the absorbance of the control, and A_sample_ is the absorbance of the sample.

### 2.7. Biological Activities (Antienzymatic Activities)

#### 2.7.1. Anti-Xanthine Oxidase Activity

The anti-xanthine activity (anti-XOD) of various extracts was assessed. All *S. undulata* samples (50 μg/mL) were tested in 96-well plates. 50 μL of extracts and 30 μL of the XOD solution (0.1 U/mL) were combined in a 96-well plate with 60 μL of sodium phosphate buffer (Na_2_HPO_4_, 70 mM, pH = 7.5). A volume of 60 μL of xanthine solution (150 M) was added after 15 min of pre-incubation at 25 °C, and the plate was then incubated for 10 min, and the absorbance was measured at 295 nm. Allopurinol at 1 µg/mL was used as a standard. The following formula was used to determine the inhibition percentage of XOD activity:

(2) $$\mathrm{XOD}\;\mathrm{inhibition}\;\left(\%\right)=\frac{A_{\mathrm{control}}-{\;A}_{\mathrm{sample}}}{A_{\mathrm{control}}}\times 100$$

where A_control_ is the absorbance of the control, and A_sample_ is the absorbance of the sample.

#### 2.7.2. Anti-5-Lipoxygenase Activity

The anti-5-lipoxygenase activity of *S. undulata* extracts has been determined according to Znat et al. [19], with modifications. A final volume of 250 μL was prepared by separately combining 20 μL of each extract with 150 μL of sodium phosphate buffer (pH 7.4), 20 μL of 5-lipoxygenase (5-LOX), and 60 μL of linoleic acid (3.5 mM). After 10 min of incubation at 25 °C, the absorbance was determined at 234 nm. The nordihydroguaiaretic acid NDGA, at 4 µg/mL, was used as a reference. The following formula was used to determine the 5-LOX activity inhibition:

(3) $$5-\mathrm{LOX}\;\mathrm{inhibition}\;\left(\%\right)=\frac{A_{\mathrm{control}}-{\;A}_{\mathrm{sample}}}{A_{\mathrm{control}}}\times 100$$

where A_control_ is the absorbance of the control, and A_sample_ is the absorbance of the sample.

#### 2.7.3. Anti-α-Glucosidase Activity

α-glucosidase inhibitory activity has been determined according to Kim et al. [20], with modifications. In this study, 50 μL of sodium phosphate buffer (0.1 M, pH 8.0), 100 μL of enzyme solution, and 50 μL of plant extract were added in a 96-well microplate reader (MultiskanTM GO Thermo Scientific), and preincubated for 10 min at 25 °C. Then 50 μL of PNPG (5 mM) was added to the mixture as a substrate, and the plate was incubated at 250 °C for 5 min. Acarbose was used as a reference, and the absorbance was measured at 405 nm. The following formula was used to determine the α-glucosidase activity inhibition:

(4) $$\alpha -\mathrm{Glucosidase}\;\mathrm{inhibition}\;\left(\%\right)=\frac{A_{\mathrm{control}}-{\;A}_{\mathrm{sample}}}{A_{\mathrm{control}}}\times 100$$

where A_control_ is the absorbance of the control, and A_sample_ is the absorbance of the sample.

#### 2.7.4. Anti-α-Amylase Activity

The anti-α-amylase activity of *S. undulata* extracts was determined using DNS, a spectrophotometry method that measures the concentration of the reducing sugars at 540 nm [21]. In an Eppendorf tube, 25 µL of enzyme solution (2 mg/mL) was added to 25 µL of various extracts and to 25 µL of plant extract (1.3 mg/mL). After incubation for 10 min at 25 °C, 1% starch solution was added to the mixture, and 3 min later the reaction of the enzyme solution was stopped by adding 50 µL of DNS solution (96 mM). The reaction mixture was placed in a boiling water bath for 10 min, followed by cooling at ambient temperature. Next, various solutions were diluted with 1 mL of distilled water. Acarbose was used as a positive control. Over time, the enzyme inhibitory activity was measured at 530 nm using a microplate reader. The following formula was used to determine the α-amylase activity inhibition:

(5) $$\alpha -\mathrm{Amylase}\;\mathrm{inhibition}\;\left(\%\right)=\frac{A_{\mathrm{control}}-{\;A}_{\mathrm{sample}}}{A_{\mathrm{control}}}\times 100$$

where A_control_ is the absorbance of the control, and A_sample_ is the absorbance of the sample.

### 2.8. Statistical Anylasis

Graphics were created using Origin Lab software. Data was done in triplicate and expressed as mean ± SD. All results were provided for the study of variance (ANOVA), accompanied by a Duncan test to assess if there were any significant differences (*p* < 0.05) between the averages. All statistical analysis and heatmaps were performed using XLSTAT 2009.

## 3. Results and Discussion

### 3.1. Extraction Yields

Extraction is a separation technique used to selectively separate one or more compounds from a mixture based on their chemical and physical properties. It is a very important step in the isolation and identification of bioactive molecules from the plant. There are several techniques for the extraction of biomolecules from plant sources. These techniques can be either conventional, also called traditional, or alternative [22]. In this study, *S. undulata* samples were extracted using a conventional (maceration) and non-conventional (ultrasound-assisted) technique. Ethanol and water were used to extract the three parts of *S. undulata* (TB, LV, and FL). The extraction yields obtained for all extracts were illustrated in Table 1. The yield of the *S. undulata* extracts ranged from 0.12 to 1.16% according to the plant parts, techniques, and solvents used. The best yield for tubers was achieved for an aqueous ultrasound extract, while the lowest was achieved for maceration (aqueous). For the leaves, maceration produced the highest yields (0.57 and 0.48%), although the lowest for ultrasound ethanolic extract. Finally, the highest yield for flowers was for the ultrasound aqueous extract and the lowest for the aqueous maceration. In most cases, the yields of the water extracts were higher than those of the ethanol, which has a lower polarity compared to the water, which indicates a difference in the chemical composition of the extract and a high content of polar compounds. The low yield of extraction was noted in other studies. It has been reported by S. Palmieri et al. [23] that on coriander seeds, the extraction yield ranged from 0.57 to 2.36% using ethanol and different extraction methods. For the hemp, the data shows that the ultrasound-assisted extraction led to a low yield (0.71%), similar to our findings for the aqueous leaf ultrasound extraction [23]. Our results are in line with data from the literature for the same techniques [24].

### 3.2. Polyphenol Content (TPC)

**Figure 1:**
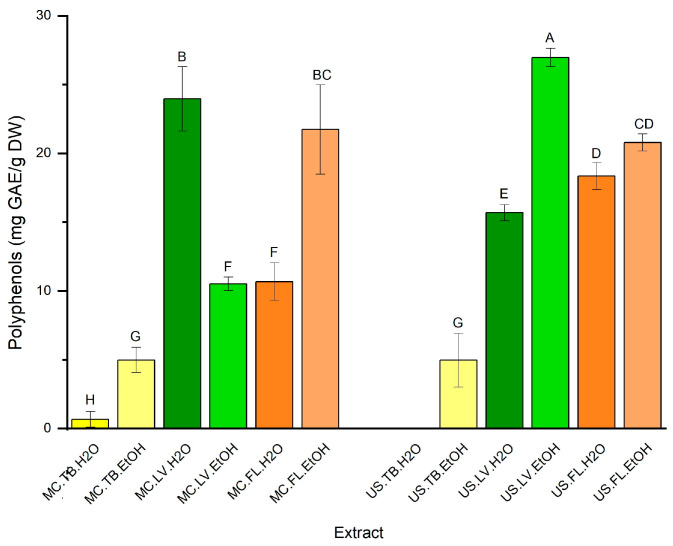
Polyphenol content of different extracts of *S.* undulata parts (MC: maceration; US: ultrasound; TB: tubers; LV: leaves; FL: flowers; H_2_O: water; EtOH; ethanol; GAE: gallic acid equivalence; DW: dry weight). Extracts were tested at 50 µg/mL. Means values ± SD (n = 3). Different letters on the histograms mean a significant difference between extracts (*p* ≤ 0.05).

The total phenolic content (TPC) of *S. undulata* extracts was determined using the calibration curve (y = 0.0057x + 0.0285 and R^2^ = 0.9998). Figure 1 shows that different plant parts, solvents, and extraction technique combinations led to a total polyphenol content ranging from 0.66 to 26.97 mg GAE/g DW from *S. undulata*. The data analysis showed a significant difference between extracts (*p* < 0.05). The highest TPC was measured in the leaf extract obtained by ultrasound extraction (26.97 mg GAE/g DW) and flowed at 23.97 mg GAE/g DW (MC-LV-H_2_O), while low TPC was observed for all tuber extracts. The total phenolic content was found in higher concentration in the aerial part (leaves (LV) and flowers (FL)) than in tuber extract (TB), which was confirmed by [25]. For the solvent, the TPC concentration is higher in ethanol than in the aqueous extract, except for the leaf extract obtained by maceration. Various researchers confirmed the results that the chemical profiles of plant extracts were dependent on the extraction method, solvent, and plant parts [25,26,27].

### 3.3. Identification of Volatile Compounds by GC-MS Analysis

**Figure 2:**
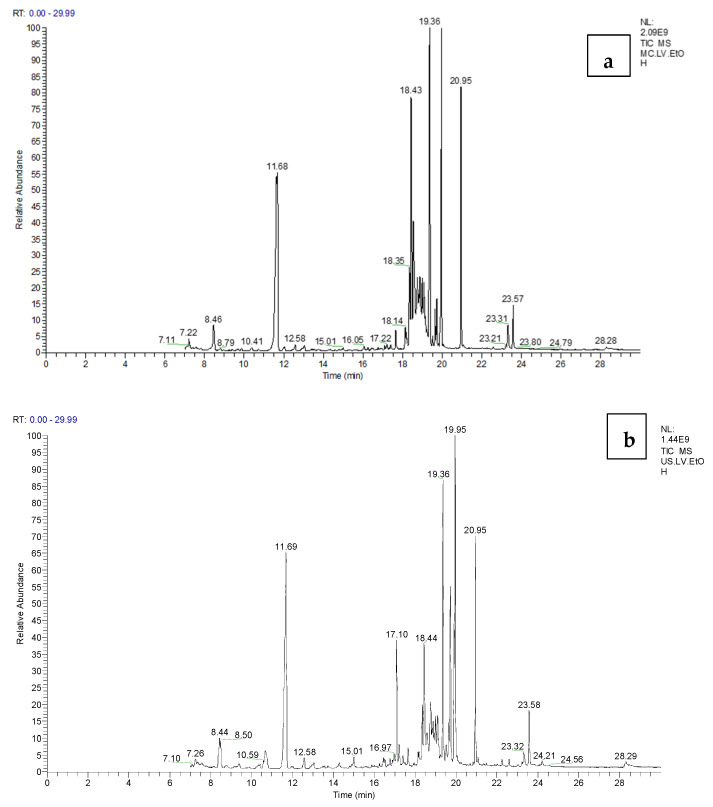
GC–MS chromatogram (Example) of volatile compounds in *S. undulata* extracts, (**a**); MC.LV.EtOH and (**b**); US.LV.EtOH.

The different extracts of *S. undulate* parts were analyzed by GC-MS (Table 2 and Table 3 and Figure 2) to determine the volatile composition. The GC-MS technique made it possible to identify 25 compounds (before and after derivatization). The volatile profile from the various extracts showed the presence of several organic compound classes such as phenols (2,4-Di-tert-butylphenol, Phenol, 2,2′-methylenebis[6-(1,1-dimethylethyl)-4-methyl-)], fatty alcohol (1-Hexadecanol, 1-Hexadecanol, 2-methyl-, 9-Hexadecenoic acid, 9-octadecenyl ester, (Z, Z), Hexanoic acid, Decanoic acid, Palmitic Acid), organic acid, (carbonic acid, lactic acid, butanedioic acid, and Malic acid), steroid (2(4H)-Benzofuranone, 5,6,7,7a-tetrahydro-4,4,7a-trimethyl-, (R)-), alkane and alkane derivates (Heneicosane, Octadecane, 1,1′-[(1-methyl-1,2-ethanediyl)bis(oxy)]bis-, Heneicosane, 11-(-ethylpropyl), Heptadecane, 9-hexyl-, Octadecane, 3-ethyl-5-(2-ethylbutyl)- ). Several compounds were in common between the extraction method (e.g., 2,4-Di-tert-butylphenol, phenol, 2,2′-methylenebis[6-(1,1-dimethylethyl)-4-methyl-]), the solvent (e.g., 2,4-Di-tert-butylphenol, α-d-Fructofuranose), and the plant parts (e.g., palmitic acid, lactic acid, and α-d-Fructopyranose). For the tubers, independently from the extraction methods, the most volatile compounds were detected in the ethanolic extract, and 2,4-Di-tert-butylphenol was found in all extracts. As previously reported by Choi et al. [28] from sweet potatoes, this compound has an antioxidant and a neuroprotective effect. Concerning the leaves, in total 15 compounds were detected by the GC-MS technique: eight compounds before derivatization and seven after derivatization. Among these compounds, four were common in all leaf extracts: 2,4-Di-tert-butylphenol, lactic acid, glycerol, and palmitic acid. The flower extracts revealed 31 volatile compounds, of which 73% were found after derivatization. Monoacetin, an element of the alkanoic acids, is one of those compounds; it acts as a humectant and emulsifier in the food, pharmaceutical, and cosmetic sectors. In addition to being a tanning agent and a component of explosives, monoacetin serves as a monomer in the creation of biodegradable polyesters [29]. Palmitic acid was common for the majority of *Scorzonera* extract; according to [30], this compound was the major chemical compound of *Carthamus* caeruleus root methanolic extract, and it may have anti-inflammatory effects, given that it demonstrated considerable inhibitory action in the enzyme kinetic analysis of phospholipase. The structure of different compounds identified by GC-MS after and before derivatization is represented in the supplementary file.

### 3.4. Identification of Phenolic Compounds by LC-MS Analysis

The results of high-performance liquid chromatography are presented in Table 4 for the two extraction methods (maceration and ultrasound). A total of twenty-one compounds were identified: eleven phenolic acids (quinic acid, 1,3-dicaffeoylquinic acid, gallic acid, protocatechuic acid, caffeic acid, synergic acid, p-coumaric acid, ferulic acid, chlorogenic acid, rosmarinic acid, cinnamic acid) and ten flavonoids (rutin, hyperoside, luteolin-7-*O*-glucoside, naringin, quercetin, apigenin, quercetin, kaempferol, naringenin, apigenin). For the maceration, the results in Table 4 reveal the presence of all twenty-one phenolic compounds distributed among the three parts (TB, LV, and FL). The tuber extracts are characterized by low levels of phenolic acids. The total concentration was estimated at 35.87 mg/100 g DM, with an abundance of quinic acid at 34.19 mg/100 g DW, while flavonoids were detected at a low concentration of 0.45 mg/100 g DW in total. Concerning the leaf extract, eight phenolic acids were detected. The highest content is observed for quinic acid at 165.55 mg/100 g DW, followed by chlorogenic acid at 131.34 mg/100 g DW. Quinic acid is a cyclohexanecarboxylic acid that can be found in the extracts of many different medicinal plant parts, such as those from *Ziziphus lotus* L., *Haematocarpus validus*, *Coffea arabica*, *Ziziphus lotus* L., *Hypericum empetrifolium*, and others, and it has a variety of biological properties, including antioxidant, anti-diabetic, and anti-cancer activity [31]. For the chlorogenic acid, Sarıaltın and Acıkara [32] confirmed the presence of this phenolic acid in both aerial parts and roots of Scorzonera extracts, with the highest amount in *S. coriacea* aerial parts (12.73 mg/g DW). Additionally, chlorogenic acid has been reported as the most vital phenolic acid for the Asteraceae family, with an anti-inflammatory and antioxidant effect [32]. For flavonoids, the leaves were characterized by ten compounds with a total concentration of 156.58 mg/100 g DW. The major flavonoids of this organ were luteolin-7-*O*-glucoside (245.11 mg/100 g DW), kaempferol (153.50 mg/100 g DW), apigenin (60.05 mg/100 g DW), and naringin (37.35 mg/100 g DW). The LC-MS patterns of the maceration show that the flower extracts have the highest content of phenolic acids, with a total concentration of 286.31 mg/100 g DW. Chlorogenic acid and quinic acid are still the major compounds (196.34 mg/100 g DM and 81.70 mg/100 g DM, respectively). In addition, the flower extracts contain eight flavonoids (154.95 mg/100 g DW), of which kaempferol, apigenin, luteolin-7-*O*-glucoside, quercitrin, and naringin are the major compounds. The methanolic extracts prepared by ultrasound extraction (Table 4) show phenolic acid contents close to those found by maceration, where the concentration was about 334.14 mg/100 g DW, 223.97 mg/100 g DW, and 43.42 mg/100 g DW, respectively, for the flowers, leaves, and roots. The major phenolic acids (quinic acid and chlorogenic acid) were generally the same for both extraction methods. The ultrasound extraction improves the flavonoid contents of the two aerial parts (322.39 and 199.50 mg/100 g DW for flowers and leaves, respectively). This may be due to a synergy between the solvent (methanol) and the effect of sound waves on flavonoid extraction. While the tuber extract still shows a low content of flavonoids (0.2 mg/100 g DW). The LC-MS pattern confirms the results of the colorimetric assay, which showed that the tuber part contained the lowest concentration of phenolic compounds. Our results confirm those found by Lendzion et al. [1] that show that both quinic and chlorogenic acids are major compounds in the majority of *Scorzonera* species, which explains their antioxidant powers. Other studies confirm the presence of major flavonoids in *Scorsonera* (quercetin, luteolin, apigenin, quercetin, and kaempferol) [25,33]. The structure of different compounds identified by LC-MS is represented in the supplementary file.

### 3.5. Antioxidant Activity

**Figure 3:**
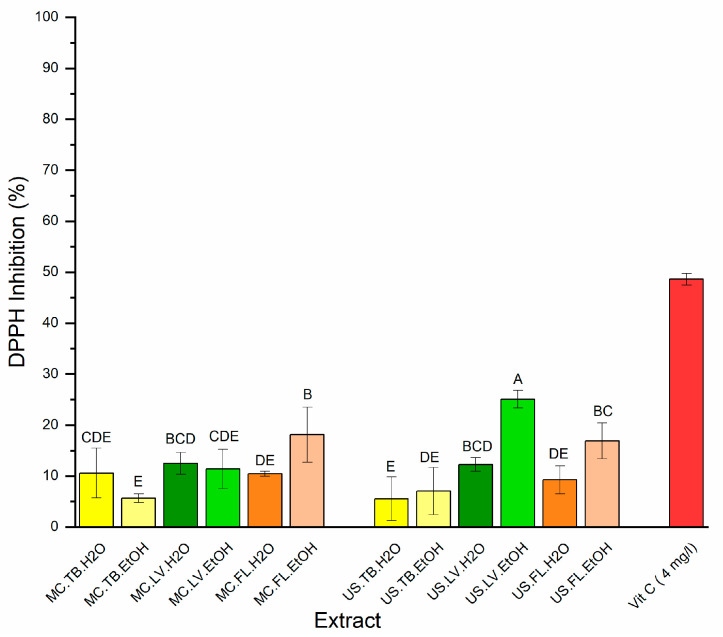
Antioxidant activity of different extracts of *S.* undulata parts (MC: maceration; US: ultrasound; TB: tubers; LV: leaves; FL: flowers; H_2_O: water; EtOH: ethanol; Vit C: vitamin C). Extracts were tested at 50 µg/mL. Means values ± SD (n = 3). Different letters on the histograms mean a significant difference between extracts (*p* ≤ 0.05).

The antioxidant activity of *S. undulata* extracts was carried out using the DPPH scavenging activity, and results are presented in Figure 3. The extract concentration was tested at 50 µg/mL, and VIT C (ascorbic acid) was tested as a reference at 4 µg/mL. The antioxidant activity of the three parts (TB, LV, and FL) varied from 5.55% for the tubers ultrasound aqueous extract to 25.06% for the leaves ultrasound ethanolic extract. The extracts showed a pattern that was comparable to that of total phenolic content, where, in general, the aerial part and the ethanolic extracts showed the highest antioxidant activity. This could be explained by the positive correlation between the total polyphenolic content (TPC) and the antioxidant activity of the extracts. These results were in accordance with those reported for three *Scorzonera* species, indicating that the aerial parts of *S.* pygmaea showed a high antioxidant capacity, which was correlated with its total polyphenolic content [34]. It was noted in this study that the tubers of *Scorzonera undulata* have lower antioxidant activity, regardless of the solvents (water and ethanol) and the extraction methods (maceration and ultrasound) that were used. It can be explained by the fact that the tubers represent the organ reserve of *S.* undulata. The reserve carbohydrate in the Asteraceae family is inulin. It’s a linear polysaccharide [35]. Due to its advantageous effects on gastric health, inulin is frequently used as a prebiotic, fat and sugar replacer, texture modifier, and for the creation of functional meals in order to promote health [36]. In the roots of *S*. *hispanica*, inulin represents 20% of the plant material [1]. The ANOVA test (*p* < 0.05) showed a statistically significant difference between the two extraction methods except for the tuber aqueous extract and the ethanolic leaf extract.

### 3.6. Biological Activities (Antienzymatic Activities)

To the best of our knowledge, the anti-xanthine oxidase, the antidiabetic activity (anti-alpha amylase, anti-alpha glucosidase), and the anti-inflammatory activity of *S. undulata* parts have not been reported in previous studies. In vitro tests, originally reported by Ben Khadher et al. [16], were used to estimate the anti-enzymatic activity of extracts (Table 5). The different extract was tested at 50 mg/L. For the xanthine oxidase activity, allopurinol (1 µg/mL) was tested as a positive control, and the result shows low activity for all the extracts (0 ≤ IP ≤ 38.26). The highest inhibition activity was detected for the leaf ethanolic extract obtained by maceration. This can be explained by the high amount of luteolin-7-*O*-Glucoside, which was identified as an inhibitor of Xanthine Oxidase in the *Flos Chrysanthemum* extract [37]. All tubers and extracts were inactive against xanthine oxidase. It can be explained by the fact that the *S.* undulata roots are devoid of the bioactive molecules responsible for this activity, such as flavonoids and their compounds (quercetin, rutin, naringenin, etc.), hydroxycinnamic acids (chlorogenic acid, dihydrocaffeic acid, 4-*O*-caffeoylquinic acid, etc.), tannins, and others [38]. The 5-lipoxygenase was used as an enzyme to evaluate the inflammatory activity. The 5-LOX is an enzyme that is considered to be a reason for many disorders, such as inflammatory disorders (rheumatoid arthritis and allergic rhinitis) and asthma [39]. The nordihydroguaiaretic acid (NDGA) was used as a reference for the anti-inflammatory activity. The activity levels of *S.* undulata ranged from 0% for most of the extracts to 14.05% for the ethanolic leaf extract obtained by ultrasound extraction. Our anti-inflammatory results are in harmony with those found by [40], who found that the essential oil of *Lantara Camara* showed low anti-inflammatory activity (IC_50_ = 81.5 µg/mL). The antidiabetic activity of *Scorzonera* undulata extracts was tested using two enzymatic assays: anti-alpha glucosidase and anti-alpha amylase activities, and acarbose (50 µg/mL) was tested as a reference. Our result showed lower activity against alpha glucosidase; the inhibition percentage ranged from 0 to 9.77% for the aqueous flower extract obtained by ultrasound. However, for the alpha-amylase, the inhibition percentage at 50 µg/L ranged from 0 to 31.34% for the aqueous tuber extract obtained by maceration. Because this is the first study to be evaluated, comparisons cannot be made with previous results.

### 3.7. Statistical Anylasis

#### 3.7.1. Correlation Coefficient Analysis

**Figure 4:**
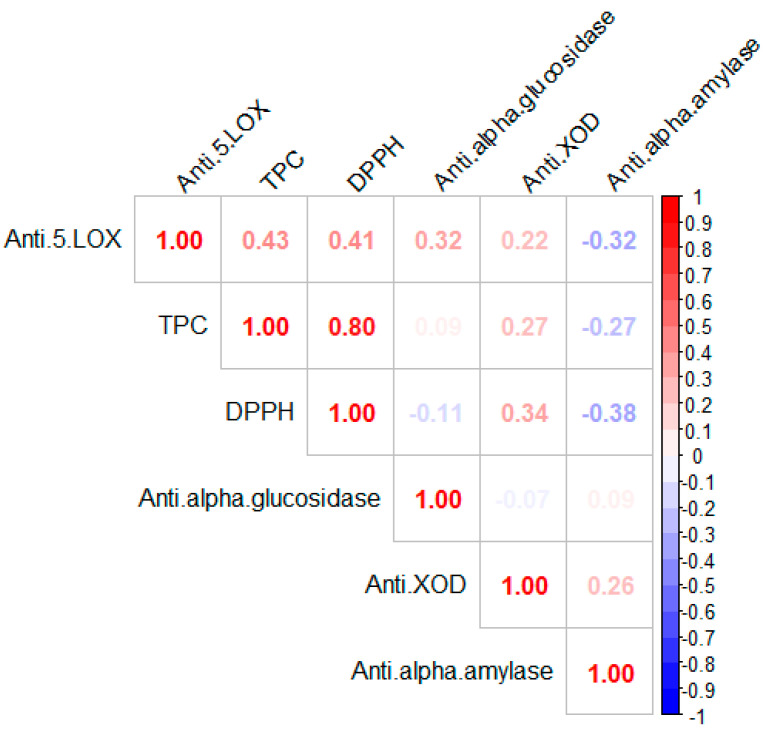
Correlation coefficients of total phenolic contents, DPPH, and biological activities from *S. undulata* extracts using Pearson correlation. Different R values of Pearson correlations are represented by various square colors.

The correlation between total polyphenol content (TPC), antioxidant (DPPH), and anti-enzymatic activities (antidiabetic (alpha-amylase and alpha-glucosidase), anti-inflammatory (5-LOX), and anti-xanthine oxidase (Anti-XOD)) were analyzed. According to the data represented in Figure 4, the Pearson correlation study revealed both positive and negative correlations. The correlation coefficient was 0.80 between TPC and DPPH. The correlation coefficient between polyphenol content and antioxidant activity in plant extracts has been the subject of several studies. In this regard, Maina, S. [41] noticed a significant correlation between TPC and DPPH (R^2^ = 0.96) of *Cleome* gynandra L. Similarly, a study by Sarker, U. et al. [42] confirms the strong correlation between TPC and DPPH. Furthermore, we observed a moderately positive correlation between TPC and anti-5-LOX, DPPH and anti-5-LOX, anti-alpha glucosidase, and anti-5-LOX, anti-XOD and anti-5-LOX, TPC and anti-XOD, DPPH and anti-XOD, anti-alpha amylase and anti-XOD at R^2^ = 0.43, 0.41, 0.32, 0.22, 0.27, 0.34, and 0.26, respectively. Conversely, strongly negative correlations were found for the anti-alpha amylase activity with anti-5-LOX, TPC, and DPPH. Our findings were in accordance with the data obtained from Wang, L. et al. [43].

#### 3.7.2. Heat Map Analysis

**Figure 5:**
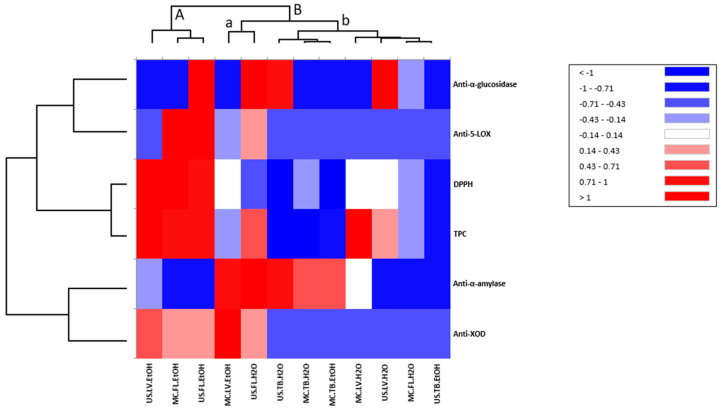
Heatmap for total phenolic contents, DPPH, and biological activities of S. undulata extracts. Extracts are presented on the horizontal axis, red color presents the highest levels of total polyphenols (TPC), antioxidant (DPPH), antidiabetic (alpha-amylase and alpha-glucosidase), anti-inflammatory (5-LOX) and anti-xanthine oxidase (Anti-XOD) activities. A, B, a and b different groups obtained after heatmap analysis.

Based on their polyphenol content, antioxidants, and biological activities, extracts are represented in the heatmap (Figure 5). The *S. undulata* extracts can be clearly separated into two principal groups (A and B). The first group A contains three extracts (US.LV.EtOH, MC.FL.EtOH, and MC.FL.EtOH), and the second group B contains the other extract. Interestingly, the three extracts from group A are characterized by the highest polyphenol content, DPPH radical scavenging activity, and anti-xanthine activity. Furthermore, the positive correlation between the TPC and DPPH was clearly represented in group A (red color). Moreover, a high positive correlation between total polyphenol content and DPPH activity of tea was found by Dobrinas, S. et al. [44]. Group B can be divided into two other groups (a and b); the first group a contained only two extracts (MC.LV.EtOH and US.FL.H_2_O), which were characterized by low anti-xanthine oxidase and anti-alpha amylase activities. The other seven extracts were regrouped into group B, with a blue color predominance. The other seven extracts were regrouped into group B with a blue color predominance. This cluster indicated lower polyphenolic content and antienzymatic activity in this group.

## 4. Conclusions

To assess the bioactive composition of the specie *Scorzonera undulata* collected from southern Tunisia, a chromatographic analysis (GC-MS and LC-ESI-MS) was used to determine the volatile and phenolic composition of three parts of *S. undulata*. Its antioxidant potential and biological properties were evaluated. In total, twenty-one phenolic molecules were quantified in different extracts: eleven phenolic acids and ten flavonoids. The predominant compounds were quinic acid, chlorogenic acid, kaempferol, apigenin, luteolin-7-*O*-glucoside, quercitrin, and naringin. Twenty-five volatile compounds have been detected by GC-MS. Both aerial parts showed considerable antioxidant and biological properties compared to the tuber due to their richness in phenolic compounds. This study shows the difficulties in establishing a single extraction method and solvent that is effective for all the plant parts and highlights the significant variations between the three *S. undulata* parts under investigation.

## Figures and Tables

**Table 1:** Extraction yields of *S. undulata* part extracts (% DW).

	Water			Ethanol		
	TB	LV	FL	TB	LV	FL
Maceration	0.81	0.57	0.12	0.16	0.14	0.19
Ultrasound	1.16	0.47	0.29	0.14	0.12	0.15

TB: Tubers; LV; Leaves; Flowers: FL.

**Table 2:** Identification of volatile compounds by GC-MS, before derivatization, of the extracts of *S. undulata* parts.

N°	Compound Name	RT	MF	MW	Maceration						Ultrasound					
					H_2_O			Ethanol			H_2_O			Ethanol		
					TB	LV	FL	TB	LV	FL	TB	LV	FL	TB	LV	FL
1	1-Hexadecanol	14.64	C_16_H_34_O	242.4						X						
2	2,4-Di-tert-butylphenol	16.33	C_14_H_22_O	206.3	X	X		X	X		X	X	X	X	X	X
3	10-Heneicosene	16.41	C_21_H_42_	294.6										X		
4	1-Hexadecanol, 2-methyl-	16.42	C_17_H_36_O	256.5											X	
5	Octadecane,1,1′-[(1-methyl-1,2 ethanediyl)bis(oxy)]bis-	17.52	C_39_H_80_O_2_	581.1								X				
6	2(4H)-Benzofuranone,5,6,7,7a-tetrahydro-4,4,7a-trimethyl-, (R)-	17.64	C_11_H_16_O_2_	180.2					X							
7	Phenol,2,2′-methylenebis[6-(1,1-dimethylethyl)-4-methyl-	17.91	C_23_H_32_O_2_	340.4	X	X		X		X						X
8	7,9-Di-tert-butyl-1-oxaspiro(4,5)deca-6,9-diene-2,8-dione	21.07	C_17_H_24_O_3_	276.3												
9	3,7,11,15-Tetramethyl-2-hexadecen-1-ol	22.13	C_20_H_40_O	296.5											X	
10	9-Hexadecenoic acid, 9-octadecenyl ester, (Z,Z)-	28.27	C_34_H_64_O_2_	504.8									X		X	X
11	Z-10-Methyl-11-tetradecen-1-ol propionate	28.29	C_18_H_34_O_2_	282.5								X			X	
12	Heneicosane, 11-(-ethylpropyl)	19.46	C_26_H_54_	366.7						X						
13	Heneicosane	21.32	C_21_H_44_	296.5						X						
14	Pentacosane	21.33	C_25_H_52_	352.6												X

X: detected; RT: retention time; MF: molecular formula; MW: molecular weight; TB: tubers; LV: leaves; FL: flowers.

**Table 3:** Identification of volatile compounds by GC-MS, after derivatization, of the extracts of *S. undulata* parts.

N°	Compound	RT	MF	MW	Maceration						Ultrasound					
					H_2_O			Ethanol			H_2_O			Ethanol		
					TB	LV	FL	TB	LV	FL	TB	LV	FL	TB	LV	FL
1	2,3-Butanediol	7.85	C_4_H_10_O_2_	90.1										X		
2	Lactic acid	8.47	C_3_H_6_O_3_	90.0		X	X	X	X	X		X	X	X	X	X
3	Hexanoic acid	8.82	C_6_H_12_O_2_	116.1				X								
4	2-Monoacetin	10.42	C_9_H_14_O_6_	218.2		X				X		X				X
5	Glycerol	10.79	C_3_H_8_O_3_	92,0		X	X	X	X	X	X	X	X	X	X	X
6	Butanedioic acid	13.06	C_4_H_6_O_4_	118.0		X				X		X				X
7	Decanoic acid	14.90	C_10_H_20_O_2_	172,2		X					X					
8	Malic acid	15.27	C_4_H_6_O_5_	134.0			X			X						
9	α-d-Fructofuranoe	18.19	C_12_H_22_O_11_	342.3			X	X	X	X		X		X	X	X
10	α-d-Fructopyranose	18.55	C_6_H_12_O_6_	180.1			X	X	X	X						
11	Palmitic Acid	20.94	C_16_H_32_O_2_	256,4		X	X	X	X	X	X	X	X	X	X	X

X: detected; RT: retention time; MF: molecular formula; MW: molecular weight; TB: tubers; LV: leaves; FL: flowers.

**Table 4:** Phenolic acids and flavonoids from *S. undulata* extracts prepared by maceration and identified by LC–MS.

N°		*m*/*z*	MF	MW	RT	Maceration			Ultrasound		
						TB	LV	FL	TB	LV	FL
		Phenolic acid concentration (mg/100 g DW)									
1	Quinic acid	191	C_7_H_12_O_6_	192.1	1.59	34.19 ± 6.07 ^E^	165.55 ± 7.04 ^B^	81.70 ± 3.10 ^C^	40.13 ± 10.05 ^E^	187.69 ± 14.00 ^A^	63.97 ± 9.04 ^D^
2	1,3-dicaffeoylquinic acid	515	C_25_H_24_O_12_	516.4	1.61	0.28 ± 0.06 ^C^	4.41 ± 0.07 ^A^	3.40 ± 0.47 ^B^	0.48 ± 0.10 ^C^	2.97 ± 0.47 ^B^	3.08 ± 0.29 ^B^
3	Gallic acid	169	C_7_H_6_O_5_	170.1	1.64	0.05 ± 0.04 ^A^	0.14 ± 0.07 ^A^	0.09 ± 0.03 ^A^	0.09 ± 0.09 ^A^	0.18 ± 0.07 ^A^	0.12 ± 0.08 ^A^
4	Protocatechuic acid	153	C_7_H_6_O_4_	154.1	4.61	0.10 ± 0.03 ^D^	0.98 ± 0.07 ^B^	0.40 ± 0.15 ^C^	1.54 ± 0.10 ^A^	1.17 ± 0.17 ^B^	0.49 ± 0.05 ^C^
5	Chlorogenic acid	353	C_16_H_18_O_9_	354.3	8.6	1.13 ± 0.08 ^E^	131.34 ± 23.21 ^C^	196.34 ± 11.5 ^B^	1.15 ± 0.10 ^E^	31.71 ± 4.22 ^D^	254.43 ± 25.02^A^
6	Caffeic acid	179	C_9_H_8_O_4_	180.1	10.27	nd	nd	0.49 ± 0.20 ^B^	nd	nd	1.52 ± 0.09 ^A^
7	Syringic acid	197	C_9_H_10_O_5_	198.1	11.89	nd	5.28 ± 0.07 ^B^	3.86 ± 0.88 ^C^	nd	nd	10.40 ± 0.78 ^A^
8	p-coumaric acid	163	C_9_H_8_O_3_	164.1	16.2	0.02 ± 0.01 ^B^	nd	0.03 ± 0.01 ^B^	0.02 ± 0.02 ^B^	0.29 ± 0.07 ^A^	0.03 ± 0.02 ^B^
9	Ferulic acid	193	C_10_H_10_O_4_	194.1	19.54	0.03 ± 0.02 ^BC^	0.19 ± 0.07 ^A^	0.05 ± 0.01 ^BC^	0.02 ± 0.01 ^BC^	nd	0.06 ± 0.01 ^B^
10	Rosmarinic acid	359	C_18_H_16_O_8_	360.3	24.30	nd	nd	0.03 ± 0.02 ^A^	nd	nd	nd
11	Cinnamic acid	147	C_9_H_8_O_2_	148.1	29.22	0.15 ± 0.06 ^B^	0.51 ± 0.07 ^A^	nd	nd	nd	nd
			
1	Rutin	609	C_27_H_30_O_16_	610.5	21.66	0.06 ± 0.02 ^B^	0.33 ± 0.09 ^A^	nd	0.02 ± 0.00 ^C^	0.17 ± 0.07 ^B^	0.27 ± 0.09 ^A^
2	Quercetin	301	C_15_H_10_O_7_	302.2	29.12	nd	0.19 ± 0.07 ^A^	nd	nd	nd	nd
3	Hyperoside	463	C_21_H_20_O_12_	464.3	21.71	0.07 ± 0.03 ^E^	0.93 ± 0.09 ^A^	0.45 ± 0.04 ^C^	0.04 ± 0.04 ^E^	0.23 ± 0.07 ^D^	0.80 ± 0.02 ^B^
4	Luteolin-7-*O*-Glucoside	447	C_21_H_20_O_11_	448.3	22.32	0.18 ± 0.02 ^D^	245.11 ± 22.35 ^A^	31.29 ± 0.47 ^C^	nd	32.84 ± 6.22 ^C^	63.32 ± 4.22 ^B^
5	Naringin	579	C_27_H_32_O_14_	580.5	23.94	0.18 ± 0.06 ^C^	37.35 ± 15.02 ^A^	10.01 ± 0.88 ^BC^	nd	5.66 ± 0.66 ^BC^	12.67 ± 1.33 ^B^
6	Quercetrin	447	C_15_H_10_O_7_	302.2	24.47	0.11 ± 0.1 ^D^	5.91 ± 1.12 ^C^	22.33 ± 2.44 ^B^	0.15 ± 0.10 ^D^	2.91 ± 0.40 ^CD^	43.96 ± 4.55 ^A^
7	Apigenin	431	C_15_H_10_O_5_	270.0	24.58	0.06 ± 0.08 ^D^	60.05 ± 2.47 ^A^	43.35 ± 8.54 ^B^	0.01 ± 0.00 ^D^	7.61 ± 1.01 ^C^	62.40 ± 4.88 ^A^
8	kaempferol	285	C_15_H_10_O_6_	286.2	29.22	nd	153.49 ± 5.22 ^A^	46.01 ± 14.20 ^C^	nd	145.90 ± 2.33 ^AB^	135.62 ± 15.54 ^B^
9	Naringenin	271	C_15_H_12_O_5_	272.2	31.39	0.03 ± 0.02 ^D^	0.18 ± 0.07 ^C^	0.13 ± 0.03 ^CD^	0.06 ± 0.04 ^D^	0.70 ± 0.07 ^A^	0.37 ± 0.09 ^B^
10	Apigenin-7-*O*-glucoside	269	C_21_H_20_O_10_	432.4	31.95	0.01 ± 0.00^E^	1.79 ± 0.10 ^C^	1.40 ± 0.12 ^D^	0.01 ± 0.01 ^E^	3.47 ± 0.12 ^A^	2.99 ± 0.47 ^B^

RT: retention time; MF: molecular formula; MW: molecular weight; TB: tubers; LV: leaves; FL: flowers; nd: not detected. Different letters on the same row mean a significant difference between extracts (*p* ≤ 0.05).

**Table 5:** Biological activities of *S. undulata* extracts, tested at 50 µg/mL.

			Anti-XOD	Anti-5-LOX	Anti-α-Glucosidase	Anti-α-Amylase
Extract						
Maceration	H_2_O	TB	nd	nd	nd	18.42 ± 5.12 ^BC^
		LV	nd	nd	nd	12.11 ± 0.25 ^CD^
		FL	nd	nd	2.49 ± 1.96 ^B^	nd
	Ethanol	TB	nd	nd	nd	17.24 ± 4.58 ^BC^
		LV	38.26 ± 0.79 ^A^	1.23 ± 3.43 ^B^	nd	20.50 ± 0.56 ^B^
		FL	9.44 ± 7.68 ^B^	11.39 ± 1.03 ^A^	nd	nd
Ultrasound	H_2_O	TB	nd	nd	7.24 ± 2.06 ^AB^	20.08 ± 0.04 ^B^
		LV	1.65 ± 0.62 ^C^	nd	8.71 ± 2.30 ^A^	nd
		FL	11.72 ± 5.67 ^B^	3.48 ± 1.74 ^B^	9.77 ± 4.10 ^A^	31.34 ± 7.50 ^A^
	Ethanol	TB	nd	nd	nd	nd
		LV	14.05 ± 2.43 ^B^	nd	nd	8.52 ± 1.28 ^D^
		FL	11.05 ± 2.37 ^B^	14.05 ± 5.81 ^A^	9.71 ± 3.88 ^A^	nd
Reference			Allopurinol (1 µg/mL)	NDGA (4 µg/mL)	Acarbose (50 µg/mL)	Acarbose (50 µg/mL)
			52.26 ± 8.56	44.08 ± 5.30	60.22 ± 2.50	32.24 ± 7.23

TB: tubers; LV: leaves; FL: flowers; H_2_O: water; EtOH: ethanol; NDGA: nordihydroguaiaretic acid. Extracts were tested at 50 µg/mL. Means values ± SD (*n* = 3). Different letters on the same line mean a significant difference (*p* ≤ 0.05) between extracts. RT: retention time; DW: dry weight; nd: not detected.

## Data Availability

The study did not report any data.

## Supplementary Materials

The following supporting information can be downloaded at: https://www.mdpi.com/article/10.3390/life13040904/s1, Figure S1: Structure of compounds identified in *S. undulata* extract by GC-MS before (a) and after (b) derivatization; Figure S2: Structure of compounds identified in *S. undulata* extract by LC-MS.
