# Supplementary material for: Influence of Extraction Techniques and Solvents on the Antioxidant and Biological Potential of Different Parts of Scorzonera undulata
Source: Life (Basel). 2023 Mar 29;13(4):904. doi: 10.3390/life13040904 (PMC10140856; doi:10.3390/life13040904)
Supplement: Supplementary file 1 [file life-13-00904-s001.zip › life-2275471-supplementary.pdf]

Article

Influence of Extraction Techniques and Solvents on the Antioxidant and Biological Potential of Different Parts of *Scorzonera undulata*

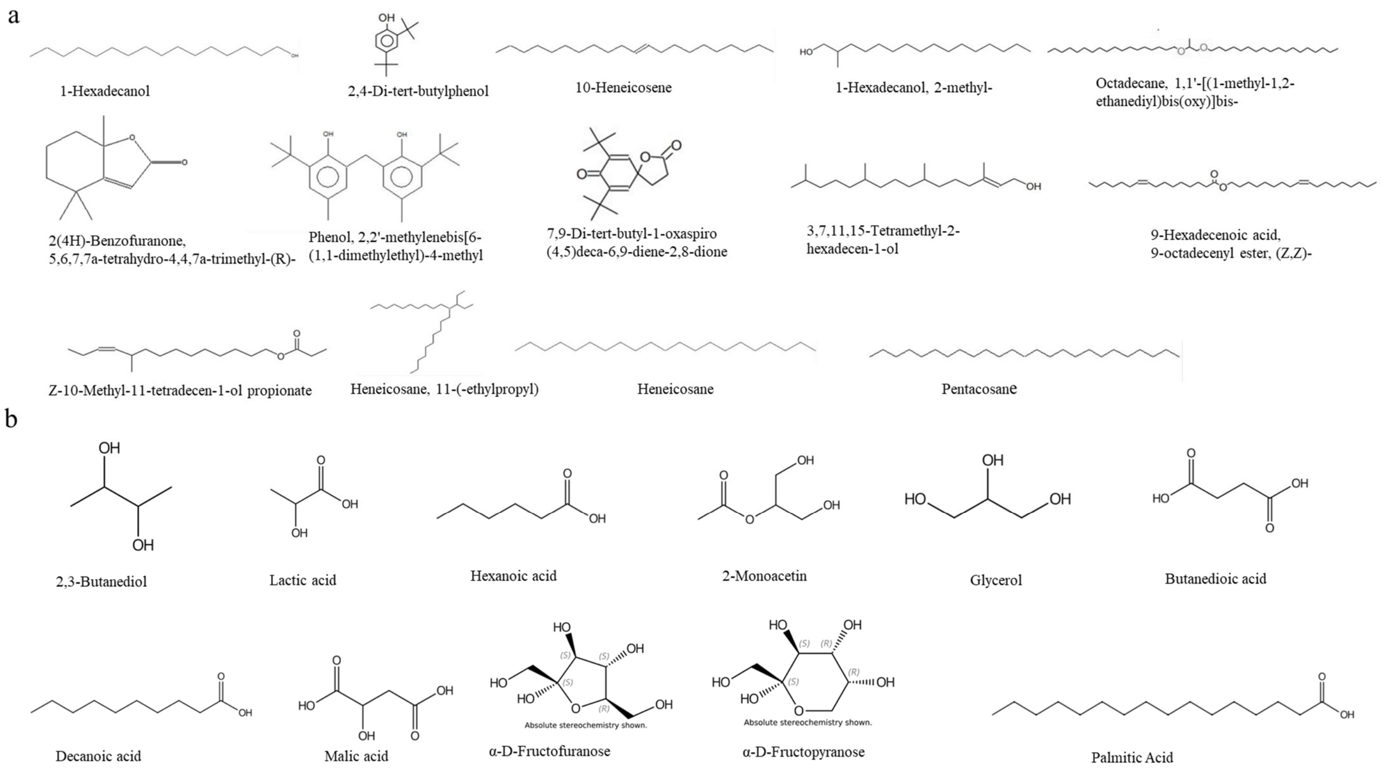

Supplementary Figure S1: Structure of compounds identified in *S. undulata* extract by GC-MS before (a) and after (b) derivatization.

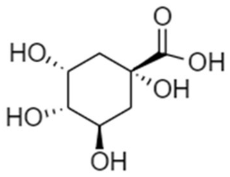

Quinic acid

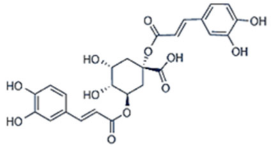

1,3-dicaffeoylquinic acid

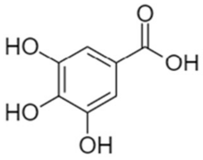

Gallic acid

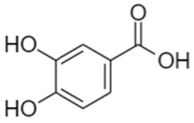

Protocatechuic acid

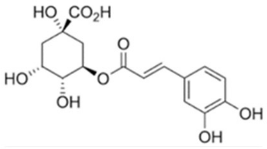

Chlorogenic acid

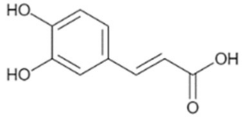

Caffeic acid

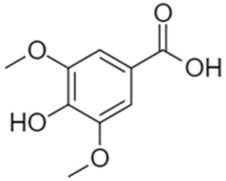

Syringic acid

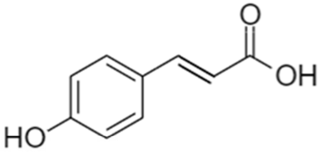

p-coumaric acid

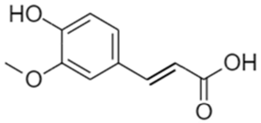

Ferulic acid

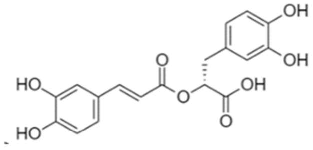

Rosmarinic acid

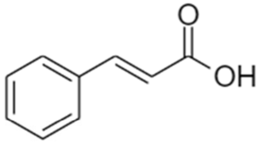

Cinnamic acid

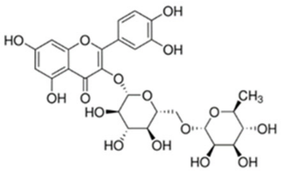

Rutin

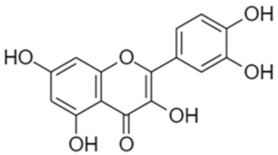

Quercetin

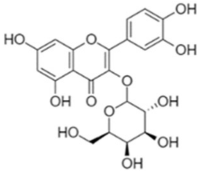

Hyperoside

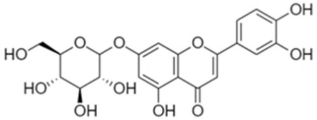

Luteolin-7-O-Glucoside

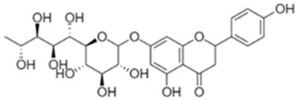

Naringin

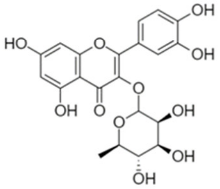

Quercetrin

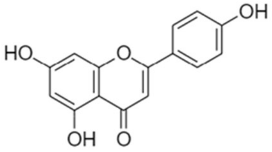

Apigenin

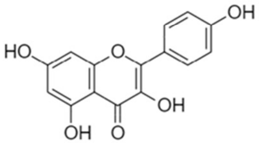

kaempferol

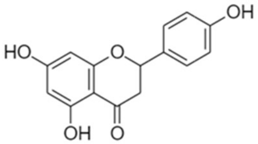

Naringenin

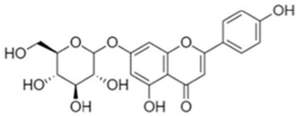

Apigenin-7-O-glucoside

Supplementary Figure S2: Structure of compounds identified in *S. undulata* extract by LC-MS.
